# Pelvic Chondrosarcoma Treated by En Bloc Resection with Patient-Specific Osteotomy Guides and Reimplantation of the Extracorporeally Irradiated Bone as an Osseocartilaginous Structural Orthotopic Autograft: A Report of Two Cases with Description of the Surgical Technique

**DOI:** 10.1155/2021/5512143

**Published:** 2021-04-02

**Authors:** Georgios Gkagkalis, Kevin Moerenhout, Hannes A. Rüdiger, Daniel A. Müller, Igor Letovanec, Stephane Cherix

**Affiliations:** ^1^Department of Orthopaedics and Trauma Surgery, Lausanne University Hospital and University of Lausanne, Lausanne, Switzerland; ^2^Department of Orthopaedics, Etablissements Hospitaliers du Nord Vaudois, Yverdon-les-Bains, Switzerland; ^3^Department of Orthopaedics, Schulthess Klinik, Zürich, Switzerland and Lausanne Sarcoma Center, Lausanne University Hospital and University of Lausanne, Lausanne, Switzerland; ^4^Department of Orthopaedics, Balgrist University Hospital, University of Zurich, Zurich, Switzerland; ^5^Institute of Pathology, Lausanne Sarcoma Center, Lausanne University Hospital and University of Lausanne, Lausanne, Switzerland; ^6^Department of Orthopaedics and Trauma Surgery, Lausanne Sarcoma Center, Lausanne University Hospital and University of Lausanne, Lausanne, Switzerland

## Abstract

Primary tumors of the pelvis are considered difficult to treat due to the complex anatomy and the proximity of important neurovascular structures. The surgical armamentarium for the treatment of these tumors has evolved with the help of cutting-edge technology from debilitating hemipelvectomies to solutions such as precise resections guided by patient-specific instruments or computer navigation and reconstruction by modular prostheses, 3D-printed custom-made implants, or orthotopic autograft reimplantation after extracorporeal irradiation. Different combinations of these techniques have been described in the literature with various rates of success. We present two cases of pelvic chondrosarcomas successfully treated by a combination of periacetabular resection with patient-specific osteotomy guides and orthotopic reimplantation of the extracorporeally irradiated autograft resulting in retention of the native hip.

## 1. Introduction

Primary tumors of the pelvis, and particularly those around the acetabulum, are difficult to treat due to the complex local anatomy, the necessity for extensive surgical approaches, and the biomechanical implications of massive bone resection. Historically, treatment of such tumors consisted in mutilating hindquarter amputations but has since evolved to limb-preserving internal hemipelvectomies or partial resections followed by complex reconstructions [1]. Enneking et al. [2] described and classified the resection zones in the pelvis as P1 (ilium), P2 (acetabulum), P3 (pubis or ischium), and P4 (ilium resection that extends into the ipsilateral sacrum). P2 or P2/3 resections are challenging from a reconstructive point of view since they involve the acetabulum. While posterior column-preserving resection techniques have been described [3], such resections usually result in disruption of the pelvic ring, which is no longer able to neutralize shear forces during gait.

Obtaining tumor-free resection margins is a principal objective in the curative treatment of sarcomas. This requires meticulous preoperative planning of the resection planes and accurate intraoperative execution of the osteotomies. Modern technologies provide the surgeon with software that allow him to virtually plan the osteotomies and with 3D-printed patient-specific osteotomy guides that accurately orientate the resection intraoperatively in order to optimize the result [4]. Allograft prosthetic composite or purely endoprosthetic reconstructive options exist, with complications ranging from inadequate soft tissue coverage of the voluminous implants and high rates of aseptic loosening to implant or allograft fractures [5]. With the onset and rapid evolution of 3D printing technology, custom-made implants have been added as valuable options to the reconstructive armamentarium of the surgeon [6–9].

Extracorporeal irradiation (ECI) of a resected bone segment and reimplantation as a structural autograft (autograft recycling) in the treatment of malignant bone tumors has been introduced in 1968 [10]. It may provide a lasting biological reconstruction, while there is currently no evidence of local recurrences within the autograft due to the application of high doses of irradiation [11].

We present two patients treated with a hip-preserving strategy of en bloc resection of the tumor with the use of patient-specific 3D-printed osteotomy guides and ECI of the specimen, which was then reimplanted in its anatomic position as a recycled osseocartilaginous autograft. To our knowledge, it is the first report of the combination of the two techniques in the treatment of a pelvic primary tumor with retention of the native hip. Informed consent regarding the use of their medical files for publication purposes was obtained from both patients.

## 2. Case Presentations

### 2.1. Case 1

**Figure 1:**
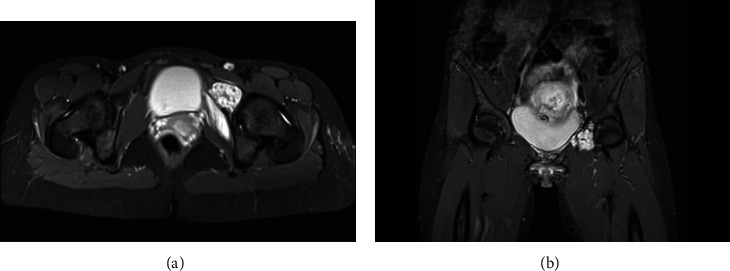
Axial (a) and coronal (b) T2 STIR weighted views on initial MRI of the pelvis showing a chondrogenic lesion of the left iliopubic ramus extending in the anterior acetabular wall.

A 30-year-old pregnant patient was referred to a local hospital with left hip pain after a low energy trauma. As she was in her 11^th^ week of pregnancy, MRI was chosen over plain films, in order to assess any traumatic lesions of her left hip or pelvis. While the MRI showed no traumatic lesion, it revealed a tumor of her left iliopubic ramus infiltrating the anterior acetabular wall, evoking a chondrogenic tumor (Figure 1). The tumor had started to invade the soft tissue on the anterior aspect of the iliopubic ramus. The patient was then referred to our sarcoma center. A surgical biopsy revealed a low-grade chondroid tumor. Given the particular situation of a pelvic low-grade chondrosarcoma in a pregnant patient, a strategy to closely follow the evolution of the tumor by repeated contrast enhancement-free MRIs and to postpone definitive treatment after delivery was proposed to the patient. The tumor showed nonsignificant progression during pregnancy, and the patient gave uncomplicated birth by C-section at 38 weeks of gestation. Postpartum thoracoabdominal CT did not reveal distant metastases.

**Figure 2:**
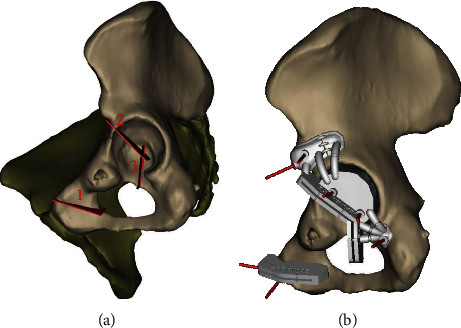
Computer-generated images demonstrating the resection lines (a) and the positioned cutting guides (b) based on MRI- and CT-based reconstructions of the patients' hemipelvis. (Images are courtesy of Materialise®, Leuven, Belgium).

Even though the biopsy revealed low-grade chondroid tissue and the tumor was anatomically accessible to curettage, the location in the pelvis and soft tissue invasion led us to propose wide en bloc excision of the tumor. The tumor infiltrated the anterior portion of the acetabulum, in a non-weight-bearing area, and no joint effusion or radiological signs of osteoarthritis were present. Approximately one third of the joint surface was affected by the resection, so that joint instability and early secondary osteoarthritis were expected in case of resection alone without reconstruction. Still, the most important two-thirds of the joint cartilage were spared, so that primary total hip arthroplasty could be avoided. For these reasons, a hip-preserving approach using custom-made osteotomy guides and ECI followed by reimplantation of the irradiated segment as massive osseocartilaginous structural graft was chosen. The surgical team planned the resection margins and the cutting guides based on MRI- and CT-based reconstructions of the patients' hemipelvis were fabricated by a company specialized in medical 3D printing (Materialise®, Leuven, Belgium) (Figure 2).

**Figure 3:**
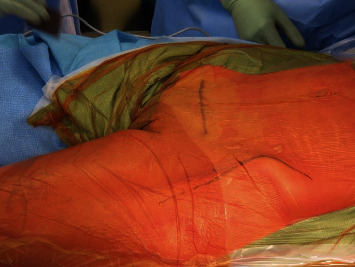
Intraoperative picture showing the planning of the incisions for the dual approach: Pfannenstiel and Smith-Petersen, respectively.

**Figure 4:**
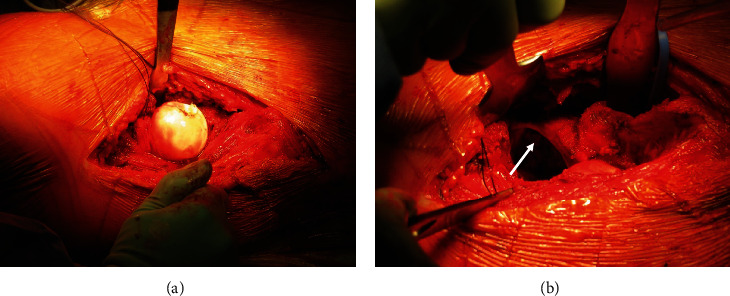
Intraoperative view of the anteriorly dislocated femoral head through the Smith-Petersen approach (a) and the acetabular cavity (b) after femoral head dislocation, demonstrating the integrity of the labrum (arrow).

**Figure 5:**
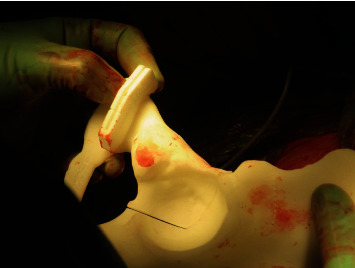
Intraoperative picture showing the verification of the position of the cutting guide on the 3D-printed model of the patient's hemipelvis.

**Figure 6:**
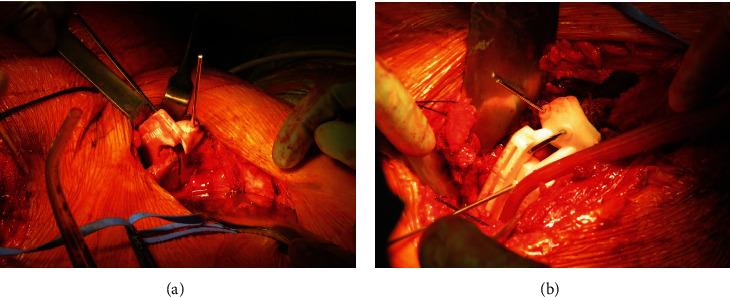
Intraoperative pictures showing the positioned cutting guide for the osteotomy of the iliopubic ramus (a) and the anterior-superior portion of the acetabulum (b).

Wide excision was performed using a double incision. A modified Smith-Petersen (Figure 3) approach was used for anterior dislocation of the hip (Figure 4) and exposure of the iliopubic eminence and the acetabulum. The previous C-section (Pfannenstiel) incision was then used to expose the iliopubic ramus. CT- and MRI-based patient-specific osteotomy guides (Figure 5) were used to perform the osteotomies through the iliopubic ramus, anterior acetabular wall, and teardrop (Figure 6). The iliopsoas muscle was preserved.

**Figure 7:**
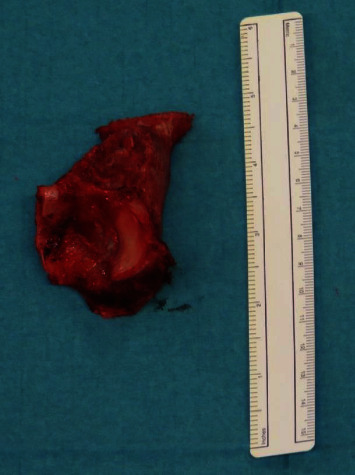
Picture of the resected bone. The tumor on the iliopubic ramus infiltrating the anterosuperior portion of the acetabulum is visible.

**Figure 8:**
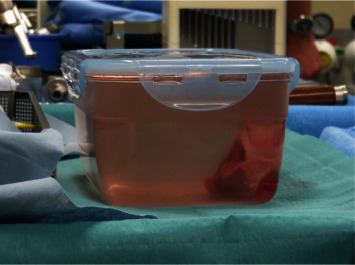
Picture of the specimen after curettage and placement in a physiologic serum filled sterile container ready to be wrapped and transported to the radio-oncology department for irradiation.

**Figure 9:**
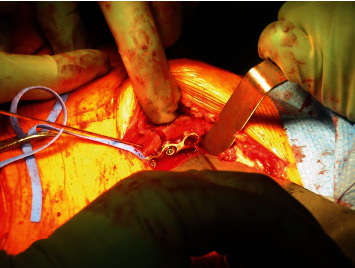
Intraoperative picture showing the plate fixation of the recycled autograft at the level of the iliopubic ramus.

Inking of the specimen was done before delicately stripping off the soft tissues from the bone surface. The material was sent to pathology for surgical margin determination together with multiple sampling of the bone at the osteotomy surface. Debulking of the tumor from the inside of the ramus and anterior wall was performed on the back table and sent to pathology in a separate container. The specimen (Figure 7) was then dived in a serum-filled box and sterile packed (Figure 8). The graft was irradiated with 10MV photons at a total dose of 50 Gy. The curetted defect in the iliopubic ramus was then filled with antibiotic-loaded cement. We added 2 g of vancomycin and 1.2 g of tobramycin per 40 ml to the 0.55 g of gentamycin-loaded cement. Finally, the irradiated bone was reimplanted in its anatomical position and fixed with plates and screws (Figure 9). Minimal gaping at the osteotomy site due to the sawblade is inevitable and was treated by compression within the fixation plates in order to maximize the probabilities of direct bone healing. No gaps were visible in the end of the procedure. Histopathological analysis confirmed a low-grade chondrosarcoma.

**Figure 10:**
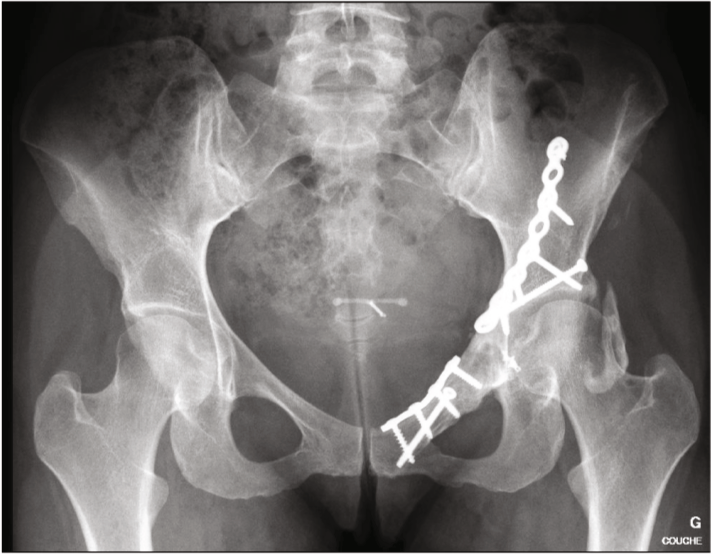
Plain film at 4 years of follow-up showing healed osteotomy sites without signs of femoral head avascular necrosis or osteoarthritis. Note the presence of heterotopic ossification.

Postoperative evolution was uneventful, and the patient was discharged home after ten days on partial weight bearing for 3 months. At 4-year follow-up, she presented full recovery of her left hip, was pain free in her daily-life activities, and had regained amateur sport activity such as skiing and jogging. Plain films revealed healed osteotomy sites and no signs of femoral head avascular necrosis or osteoarthritis (Figure 10). Pelvic MRI with metal artefact reduction sequence (MARS) showed no local nor systemic recurrence.

### 2.2. Case 2

**Figure 11:**
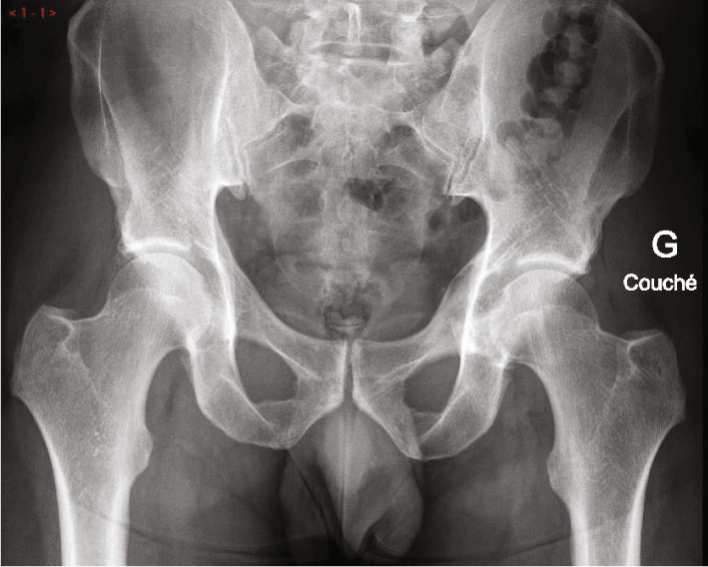
AP pelvic plain film showing a lytic lesion of the left iliopubic ramus.

**Figure 12:**
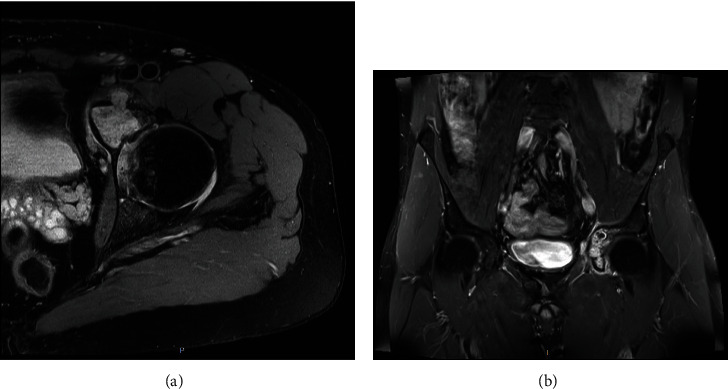
Axial (a) and coronal (b) T1 TSE sequence views on initial MRI of the pelvis showing a tumor of the left iliopubic ramus.

A 51-year-old man was investigated for bilateral knee pain in our institution's sports medicine outpatient clinic. At physical examination, minimal pain at internal rotation of the left hip was noted. A plain film of the left hip showed a lytic lesion of the iliopubic ramus (Figure 11). MRI of the pelvis revealed a chondrogenic lesion of the left iliopubic ramus extending in the anterior acetabular wall (Figure 12). Upon referral to our sarcoma center, a core needle biopsy revealed a low-grade chondroid tumor. Thoracoabdominal CT scan showed absence of metastases. Given the location of the tumor, wide resection with custom-made osteotomy guides followed by ECI and reimplantation of the irradiated segment was decided. Resection margins were planned, and patient-specific cutting guides were fabricated based on CT and MRI scans (MyOsteotomy®, Medacta, Switzerland). The same surgical technique was used as in case 1. The pathologist upgraded the initial diagnosis to a grade 2 chondrosarcoma.

**Figure 13:**
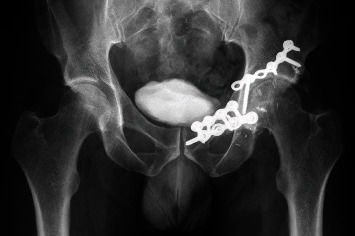
Plain film at 30 months of follow-up showing a mild thinning of the joint line.

**Figure 14:**
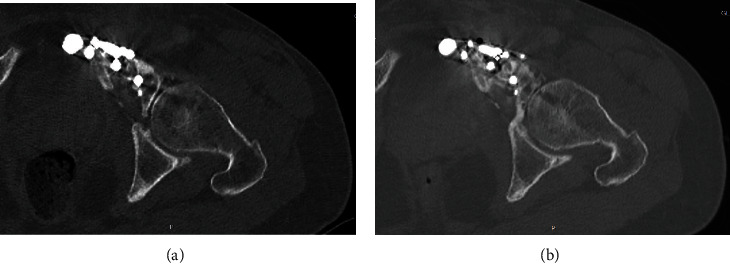
Axial view of a CT scan at 6 months (a) and at 30 months of follow-up (b) showing the healing of the osteotomy and the progression of osteoarthritis in the articular portion of the recycled autograft.

The postoperative evolution was uneventful, and the patient was discharged home 8 days after surgery. Radiological workup at six months of follow-up revealed arthritic changes of the hip in its anterior portion (Figures 13 and 14(a)). At 30 months of follow-up, plain films showed complete healing at the osteotomy sites and mild progression of the osteoarthritic changes (Figure 14(b)).

Clinically, the patient was pain free at rest but suffered from slight inguinal pain at exertion and load carrying probably due to the osteoarthritic changes at the articular portion of the recycled autograft. He worked fulltime in his prior job which involved light physical tasks. His walking distance was still unlimited, and he did not need pain medication. From the oncological point of view, the patient was disease-free.

## 3. Discussion

Primary tumors of the pelvis are rare and often difficult to treat because of the inherent complex anatomy of the pelvic girdle and the neurovascular and visceral structures in proximity. Even though low-grade cartilaginous tumors have a low incidence of local recurrence after curettage [12], there is evidence that when located in the axial skeleton they have a higher tendency to be underestimated in grade and are associated with a worse outcome than those located in the appendicular skeleton [13, 14]. Wide en bloc excision is hence the recommended option in the axial skeleton as per the staging and resection principles established by Enneking et al. [2, 15].

Whenever possible, limb salvage surgery has become the treatment of choice as new surgical techniques and reconstruction strategies have been developed [16, 17]. Massive allograft, custom-made endoprosthetic constructs [6, 8, 9] and off-the-shelf tumoral prosthesis can be used, alone or combined, in order to reconstruct the resected pelvic segment. Specific complications include aseptic loosening and inadequate soft tissue coverage [5], instability [1], infection, graft resorption, and nonunion [18].

The ECI and reimplantation of the resected bone are techniques that have been used with fair results in tumor surgery of the limbs [19–21] and the pelvis [16, 22–24]. In a recent case series, Agarwal et al. [25] presented 10 patients with a mean follow-up of 65 months treated by ECI and reimplantation for tumors involving the acetabulum. No femoral head avascular necrosis or fracture of the irradiated and reimplanted autograft was observed. In their series, patients presented a mean Musculoskeletal Tumor Society Score (MSTS) of 28/30 and no progressive arthritis was observed. Other methods of “sterilization” of the recycled autograft such as autoclaving, pasteurization, and treatment with liquid-nitrogen are reported in the literature [26, 27].

Accurate planning and execution of the osteotomies are the key to obtain tumor-free resection margins. Rapid prototyping or 3D printing technologies allow for the creation of patient-specific cutting guides that find many uses in modern orthopaedics, ranging from total joint arthroplasty to the treatment of malunions, long bone deformities, and musculoskeletal tumors with promising results [28]. Computer navigation and 3D planning have already been described in acetabular-preserving tumor resections [29–31]. In the treatment of pelvic tumors, the use of this technology can enhance the surgeons' capabilities to obtain the desired tumor-free margins without excessively large resections. There are various reports on the use of patient-specific osteotomy guides followed by reconstruction with off the shelf endoprostheses [32] or 3D-printed custom implants of the innominate bone [4, 33] and the sacrum [30]. Liu et al. [34] recently published a series of 38 patients treated for different types of sarcoma in zones 2 and 3. They allocated the patients in two groups, treated by resection with custom cutting guides and reconstruction with a modified 3D anatomic implant (group A) versus free-hand resection and reconstruction by a common 3D-printed anatomic implant (group B). They found lower operative times, less blood loss, and more accuracy in regard to the resection margins in the group where custom cutting guides were used. Higher accuracy and lower local recurrence rate were confirmed in the series published by Evrard et al. [35].

The hereby described combination of two techniques offers an association of the advantages of the different techniques when used on their own. First, the use of the patient-specific osteotomy guides maximizes the accuracy of the osteotomies. Secondly, all tumoral cells are considered eradicated in the irradiated bone, limiting thus the probability of local recurrence [19], making it safe for reimplantation from an oncological point of view. The drawback is that its biological potential (i.e., osteoinductivity) as an autograft is also reduced, and it behaves like an allograft; i.e., it has only conformal (anatomic), mechanical, and osteoconductive advantages. Another important advantage of irradiated autograft is its perfect anatomical fit, without size and conformation mismatch. Finally, the infection risk is theoretically lower than that of an allograft or of an all-endoprosthetic reconstruction or a mixed technique since a maximum of autologous tissues is preserved and there is minimal use of hardware. Moreover, in our series, we used antibiotic-loaded cement to fill the defect of the recycled autograft after curettage of the tumor, minimizing the risk of infection. Chan et al. [36] reported no infections in their series of nine patients with type 2 periacetabular resections who were reconstructed with ECI-recycled autograft and endoprosthetic replacement. Takenaka et al. [37] found a significantly high rate of complications in their series of 33 patients but their cohort consisted of autografts from various locations, like proximal humerus, proximal tibia, scapula, and only three acetabula. We believe, as they also stated in their paper, that the high heterogeneity in autograft location, method of fixation, and tumor type which also necessitated chemotherapy might have influenced the incidence and type of complications in their series.

There may also be a financial aspect to consider since the cost of the procedure that we propose is theoretically inferior to that of allograft or combined or all-endoprosthetic reconstructions, which often necessitate massive custom-made implants, although we do not have the data to support this hypothesis. Another advantage is the absence of limitations inherent to bone bank maintenance or availability in countries or institutions where allografts banks are not available [24]. Strict multidisciplinary handling protocols need to be defined in collaboration with the operating team and transport facilities to the radio-oncology department and radio-oncology medical, engineering, and technical team. The availability of a radio-oncology department in the same institution is mandatory, in order to spare time and potential process complications and delays. In our case, time from explantation to reimplantation was about one hour for both patients.

The use of osseocartilaginous pelvi-acetabular structural graft sparing the native hip eliminates the need for an immediate endoprosthetic hip reconstruction and may delay it for years, making a secondary reconstruction easier by restoring anatomy and allowing for implantation of a standard total hip prosthesis instead of a revision implant or a megaprosthesis. However, we ought to point out the fact that in both cases the tumors were located in the anterior third of the acetabulum and the weight-bearing two-thirds were considered radiologically, and proven surgically, tumor-free. This allowed the application of this native-hip sparing technique which might not be suitable for tumors located in weight-bearing portions of the acetabulum.

Altogether, potential complications of this technique are the same as structural massive allografts, including infection, nonunion of the osteotomies, fracture of the devitalized irradiated bone, and late osteoporotic changes [19]. In the two cases, we report that, at 48 and 30 months of follow-up, respectively, we did not observe any infection, hardware failure, autograft fracture, or disease recurrence. Both patients verbally claimed to be very happy with their postoperative condition and quality of life, but no official QOL evaluation was performed.

There are two important downsides to be acknowledged with the use of this method. The first is that the bone resection margins may be more difficult to analyze since only the soft tissue envelope, which has been stripped off the tumor by the surgeon in the operating room, is sent to pathology, together with the tumoral tissue that has been curetted off the bone. The second point is that the mechanical strength of the recycled autograft depends greatly on the amount of the tumor-induced bone defect. In an effort to reduce the risk of fracture, it has to be filled with bone cement as described in our technique. To our knowledge, there is no data available on these two items in the literature. Finally, avascular necrosis of the femoral head induced by the dislocation and manipulations of the proximal femur during the intervention, as well as early or delayed hip osteoarthritis, are two potential complications that are inherent to this treatment. The fact that joint cartilage is irradiated to a suppressive dose makes chondrolysis and secondary arthritic changes theoretically inevitable. Cartilage aggression and eventually destruction has also been observed in other methods of “sterilizing” bone autografts from tumoral cells, like immersion in liquid nitrogen and autoclaving as described by Hayashi et al. [38] Moreover, in case of nonanatomic reduction and fixation of the reimplanted graft, secondary osteoarthritis may develop earlier, and the risk of secondary hip replacement in an acetabulum with a nonunion may impair final results. In fact, the patient of the second case reported, developed osteoarthritic changes while the younger patient of the first reported case did not present any such changes during follow-up. We have no clear explanation to why this happened, but we assume that individual factors like age and mechanical factors such as minimal changes in articular congruity as well as individual biological response to cartilage aggression by the procedure are implicated. More data on this subject might be possible in larger series of patients treated by this method.

To our knowledge, this is the first description in the literature of treatment of a pelvic tumor by a combination of a periacetabular resection with the use of patient-specific osteotomy guides, ECI and reimplantation of the autograft with preservation of the native hip joint.

## 4. Conclusion

The combination of two techniques, namely, the use of patient-specific osteotomy guides and the extracorporeal irradiation and reimplantation of the bone as an osseocartilaginous autograft, presented satisfactory early results in the two reported cases, with the advantage of preservation of the native hip. Larger series of patients and longer follow-up are needed in order to determine if this strategy can be established as a reconstructive and therapeutic option in case of periacetabular primary pelvic tumors that do not involve the totality of the weight-bearing area.

## References

[1] Bus M. P., Szafranski A., Sellevold S. (2017). LUMiC® endoprosthetic reconstruction after periacetabular tumor resection: short-term results. *Clinical Orthopaedics and Related Research*.

[2] Enneking W. F., Spanier S. S., Goodman M. A. (1980). A system for the surgical staging of musculoskeletal sarcoma. *Clinical Orthopaedics and Related Research*.

[3] Rüdiger H. A., Dora C., Bode-Lesniewska B., Exner G. U. (2005). Extra-articular resection of the hip with a posterior column-preserving technique for treatment of an intra-articular malignant lesion. A report of two cases. *The Journal of Bone and Joint Surgery. American Volume*.

[4] Wong K. C., Kumta S. M., Geel N. V., Demol J. (2015). One-step reconstruction with a 3D-printed, biomechanically evaluated custom implant after complex pelvic tumor resection. *Computer Aided Surgery*.

[5] Hu Y. C., Huang H. C., Lun D. X., Wang H. (2012). Resection hip arthroplasty as a feasible surgical procedure for periacetabular tumors of the pelvis. *European Journal of Surgical Oncology*.

[6] Liang H. J., Ji T., Zhang Y., Wang Y., Guo W. (2017). Reconstruction with 3D-printed pelvic endoprostheses after resection of a pelvic tumour. *The Bone & Joint Journal*.

[7] Wang B., Hao Y., Pu F., Jiang W., Shao Z. (2018). Computer-aided designed, three dimensional-printed hemipelvic prosthesis for peri-acetabular malignant bone tumour. *International Orthopaedics (SICOT)*.

[8] Han Q., Zhang K., Zhang Y. (2019). Individual resection and reconstruction of pelvic tumor with three-dimensional printed customized hemi-pelvic prosthesis. *Medicine*.

[9] Wang J., Min L., Lu M. (2019). Three-dimensional-printed custom-made hemipelvic endoprosthesis for primary malignancies involving acetabulum: the design solution and surgical techniques. *Journal of Orthopedic Surgery and Research*.

[10] Spira E., Lubin E. (1968). Extracorporeal irradiation of bone tumors. A preliminary report. *Israel Journal of Medical Sciences*.

[11] Davidson A. W., Hong A., McCarthy S. W., Stalley P. D. (2005). En-bloc resection, extracorporeal irradiation, and re-implantation in limb salvage for bony malignancies. *Journal of Bone and Joint Surgery. British Volume (London)*.

[12] Brown M. T., Gikas P. D., Bhamra J. S. (2014). How safe is curettage of low-grade cartilaginous neoplasms diagnosed by imaging with or without pre-operative needle biopsy?. *The Bone & Joint Journal*.

[13] Fromm J., Klein A., Baur-Melnyk A. (2018). Survival and prognostic factors in conventional central chondrosarcoma. *BMC Cancer*.

[14] Schwab J. H., Wenger D., Unni K., Sim F. H. (2007). Does local recurrence impact survival in low-grade chondrosarcoma of the long bones?. *Clinical Orthopaedics*.

[15] Enneking W. F., Dunham W. K. (1978). Resection and reconstruction for primary neoplasms involving the innominate bone. *The Journal of Bone and Joint Surgery. American Volume*.

[16] Wafa H., Grimer R. J., Jeys L., Abudu A. T., Carter S. R., Tillman R. M. (2014). The use of extracorporeally irradiated autografts in pelvic reconstruction following tumour resection. *Bone Joint J.*.

[17] Angelini A., Calabrò T., Pala E., Trovarelli G., Maraldi M., Ruggieri P. (2015). Resection and reconstruction of pelvic bone tumors. *Orthopedics*.

[18] Bell R. S., Davis A. M., Wunder J. S., Buconjic T., McGoveran B., Gross A. E. (1997). Allograft reconstruction of the acetabulum after resection of stage-IIB sarcoma. Intermediate-term results. *The Journal of Bone and Joint Surgery. American Volume*.

[19] Poffyn B., Sys G., Mulliez A. (2011). Extracorporeally irradiated autografts for the treatment of bone tumours: tips and tricks. *International Orthopaedics*.

[20] Kotb S. Z., Mostafa M. F. (2013). Recycling of extracorporeally irradiated autograft for malignant bone tumors-long-term follow-up. *Annals of Plastic Surgery*.

[21] Barro V., Velez R., Pacha D., Giralt J., Roca I., Aguirre M. (2015). Bernese periacetabular osteotomy in a hip extra-articular resection followed by reconstruction using an extracorporeal irradiated acetabulum autograft with megaprosthesis, for proximal femur osteosarcoma in a pediatric patient. *Case Reports in Medicine*.

[22] Nishizawa K., Mori K., Saruhashi Y., Takahashi S., Matsusue Y. (2014). Long-term clinical outcome of sacral chondrosarcoma treated by total en bloc sacrectomy and reconstruction of lumbosacral and pelvic ring using intraoperative extracorporeal irradiated autologous tumor-bearing sacrum: a case report with 10 years follow-up. *The Spine Journal*.

[23] Krieg A. H., Mani M., Speth B. M., Stalley P. D. (2009). Extracorporeal irradiation for pelvic reconstruction in Ewing’s sarcoma. *Journal of Bone and Joint Surgery. British Volume (London)*.

[24] Goodwin M. L., Gundavda M. K., Reddy R. (2019). Extracorporeal radiation and reimplantation: a safe and viable option for reconstruction after sacral tumor resection?. *Annals of translational medicine*.

[25] Agarwal M. G., Gundavda M. K., Gupta R., Reddy R. (2018). Does extracorporeal irradiation and reimplantation after acetabular resections result in adequate hip function? A preliminary report. *Clinical Orthopaedics and Related Research*.

[26] Guo X., Li X., Liu T., Shuai C., Zhang Q. (2017). Pasteurized autograft reconstruction after resection of periacetabular malignant bone tumours. *World Journal of Surgical Oncology*.

[27] Li D., Li P., Ma H. (2019). Extraperiosteal segmental excision for osteofibrous dysplasia of tibia with reconstruction by liquid nitrogen-treated recycled autograft. *Journal of Orthopaedic Science*.

[28] Angelini A., Trovarelli G., Berizzi A., Pala E., Breda A., Ruggieri P. (2019). Three-dimension-printed custom-made prosthetic reconstructions: from revision surgery to oncologic reconstructions. *International Orthopaedics*.

[29] Abe K., Yamamoto N., Hayashi K. (2018). The usefulness of wide excision assisted by a computer navigation system and reconstruction using a frozen bone autograft for malignant acetabular bone tumors: a report of two cases. *BMC Cancer*.

[30] Jentzsch T., Vlachopoulos L., Fürnstahl P., Müller D. A., Fuchs B. (2016). Tumor resection at the pelvis using three-dimensional planning and patient-specific instruments: a case series. *World Journal of Surgical Oncology*.

[31] Lam Y. L., Yau R., Ho K. W., Mak K. L., Fong S. T., So T. Y. (2017). Is it possible and safe to perform acetabular-preserving resections for malignant neoplasms of the periacetabular region?. *Clinical Orthopaedics and Related Research*.

[32] Blakeney W. G., Day R., Cusick L., Smith R. L. (2014). Custom osteotomy guides for resection of a pelvic chondrosarcoma. *Acta Orthopaedica*.

[33] Chen X., Xu L., Wang Y., Hao Y., Wang L. (2016). Image-guided installation of 3D-printed patient-specific implant and its application in pelvic tumor resection and reconstruction surgery. *Computer Methods and Programs in Biomedicine*.

[34] Liu X., Liu Y., Lu W. (2019). Combined application of modified three-dimensional printed anatomic templates and customized cutting blocks in pelvic reconstruction after pelvic tumor resection. *The Journal of Arthroplasty*.

[35] Evrard R., Schubert T., Paul L. (2019). Resection margins obtained with patient-specific instruments for resecting primary pelvic bone sarcomas: a case-control study. *Orthopaedics & Traumatology: Surgery & Research*.

[36] Chan L. W., Imanishi J., Ngan S. Y. (2016). Extracorporeal irradiation and reimplantation with total hip arthroplasty for periacetabular pelvic resections- a review of 9 cases. *Sarcoma*.

[37] Takenaka S., Araki N., Ueda T. (2020). Clinical outcomes of osteoarticular extracorporeal irradiated autograft for malignant bone tumor. *Sarcoma*.

[38] Hayashi K., Yamamoto N., Takeuchi A. (2020). Clinical course of grafted cartilage in osteoarticular frozen autografts for reconstruction after resection of malignant bone and soft-tissue tumor involving an epiphysis. *Journal of Bone Oncology*.

